# Non-Viral Gene Delivery Systems

**DOI:** 10.3390/pharmaceutics13040446

**Published:** 2021-03-26

**Authors:** Henrique Faneca

**Affiliations:** CNC—Center for Neuroscience and Cell Biology, University of Coimbra, 3004-517 Coimbra, Portugal; henrique@cnc.uc.pt

The advances in the field of gene therapy have significantly improved the possibility for nucleic acids as highly promising agents for the treatment of both inherited and acquired human diseases. Substantial progress has been made in the development of different types of nucleic acids, including plasmid DNA, mRNA, microRNA, small interfering RNA, and antisense oligonucleotides. Nevertheless, despite the immense pharmacological potential of these molecules, the successful clinical application of genetic material-based strategies remains dependent on the generation of safe and effective delivery systems that have the ability to overcome the numerous biological barriers associated with the gene delivery process. 

Up to now, the large majority of gene therapy clinical trials have been based on the use of viral vectors, namely due to features such as high levels of transduction, or the efficient and stable integration of exogenous DNA into the host genome. However, several drawbacks have been associated with viral vectors, such as immunogenicity, carcinogenesis, the size limit of exogenous DNA, and the difficulty of large-scale production. Non-viral gene delivery systems have the potential to overcome these limitations, allowing not only a safe but also efficient gene delivery process into the target cells.

The aim of this Special Issue was to highlight the current progress in non-viral gene delivery systems. Six original papers and six review articles were published.

The publication by Guan and Chen et al. [1] focused on the development of the cationic human serum albumin (CHSA)-based gene delivery system, containing nuclear localization sequences (NLS), in order to overcome some of the major limitations associated with the gene delivery process, such as toxicity, immunogenicity and low transfection efficiency. The generated nanosystems (CHSA/NLS/pDNA) presented low immunogenicity and high pDNA protection against degradation by nuclease in the blood circulation and clearance by the RES system in vivo. Moreover, the nanocarriers did not exhibit significant cytotoxicity and showed great cellular uptake and gene expression efficiency promoted by the nuclear localization signal. More notably, CHSA/NLS/p53 presented substantial in vivo antitumor activity. 

Martínez-Negro and Junquera and coworkers [2] developed new non-viral gene nanocarriers composed of a cationic lipid consisting of a lysine-derived moiety linked to a C12 chain (LYCl), and a helper lipid (DOPE), and evaluated them as potential carriers to deliver two plasmid DNAs (encoding GFP and luciferase) into target cells. The computational MD calculations and the experimental data showed that the best LYCl/DOPE lipid bilayers were stable and promoted an efficient stabilization and compaction of the DNA through lipoplexes formation. These lipoplexes were structured in a Lα lamellar lyotropic liquid crystal phase consisting of a sandwich-type configuration, with alternating bilayers of LYCl/DOPE (~4.5 nm width) and pDNA and counterions monolayers (~2 nm width). The best formulation (composed by 20% of LYCl) presented high transfection efficiency and reduced cytotoxicity.

On the other hand, Saher and Zain et al. [3] evaluated the effect of 18 different sugar and polymer excipients on the performance of peptide-dendrimer/lipid/oligonucleotide nanocarriers to deliver single-stranded splice-switching oligonucleotide, under serum conditions, in different cell lines. The obtained data showed that the splice-switching activity of the formulations was significantly improved by the presence of excipients, up to 95-fold, without causing cytotoxicity. This improvement highlighted the higher impact of polymers (namely Polyvinyl derivatives) compared to sugars on transfection activity. The physicochemical characterization demonstrated that the best excipients reduced the mean diameter and surface charge of the formulations. Moreover, the type of excipient also affected the in vivo biodistribution of the formulations. 

Ibaraki and Seta et al. [4] used a nanosystem prepared with polyethylene glycol-polycaprolactone containing the peptide CH2R4H2C covalently linked (MPEG-PCL-CH2R4H2C) to efficiently deliver anti-RelA siRNA (siRelA) into tumor cells and to inhibit metastasis in tumor-bearing mice. The obtained results showed that the developed siRelA/MPEG-PCL-CH2R4H2C nanomicelles presented a high cellular uptake and inhibited the migration/invasion of B16F10 cells. Furthermore, the authors demonstrated, in a lung metastasis animal model, that the intravenous administration of siRelA/MPEG-PCL-CH2R4H2C nanosystems resulted in a high accumulation at metastatic sites and in a substantial reduction in the number of lung nodules when compared to that obtained with naked siRelA or siControl/MPEG-PCL-CH2R4H2C treatments. 

Nagachinta and de la Fuente et al. [5] aimed to generate biocompatible sphingomyelin (SNs)-based nanosystems for miRNAs delivery into colorectal cancer cells. The authors used two different strategies: the adsorption of miRNAs to the surface of cationic SNs, in SNs-ST-miRNA nanocarriers; and the precomplexation of miRNAs with the cationic lipid DOTAP for subsequent encapsulation into SNs (SNs-DOTAP-miRNA). Although both nanosystems presented the capability to carry and release miRNA145 into cancer cells, SNs-DOTAP-miRNA revealed higher transfection activity, restituting miRNA145 levels efficiently and promoting a greatest antitumor activity. 

The potential of cell penetrating peptides (CPPs), also recognized as protein transduction domains (PTDs), to be used as non-viral gene delivery systems was the focus of the review article by Taylor and Zahid [6]. In this manuscript they presented a comprehensive revision, regarding synthetic and naturally existing peptides, starting with their history, classification system, and mechanism of transduction, followed by a summary on the application of CPPs to mediate the different gene therapy strategies involving the diverse types of genetic material. 

Roma-Rodrigues and Fernandes et al. [7] provided a critical overview of the current advances in the development and use of nanotechnology-based systems to mediate antitumor gene therapy strategies. The authors presented a revision of the diverse gene therapy approaches devoted to cancer and of the different types of nanosystems, involving inorganic-, organic- and biological-based ones, that were in development towards future clinical use or already clinically applied in cancer gene therapy strategies.

On the other hand, Sato and Nakamura et al. [8] reviewed a different topic associated with the use of non-viral gene delivery systems, motivated by their low levels of transfection and their practically inexistent long-term expression when compared with viral vectors. These evidences are partially due to the fact that non-viral systems are usually used to deliver genetic material into target cells that will not be chromosomally integrated. In their manuscript, the authors presented a revision of the recent achievements involving *piggy*Bac (PB) transposon, one of three transposon systems (PB, sleeping beauty (SB) and Tol2), namely the diverse roles of PB in gene delivery and strategies to improve its performance. This work highlights the potential of PB, which promotes the chromosomal integration of the delivered transgenes, to overcome the referred limitations associated with non-viral gene delivery systems.

Carvalho, Cordeiro and Faneca [9] presented an extensive revision on the progress made in the design, generation and use of silica-based gene delivery systems, from their initial steps until the most recent developments involving their ability to mediate therapeutic strategies. The current advances in silica-based systems designed for gene therapy were reviewed, including their main properties, fabrication methods, composition versatility, diversity of structures, surface modifications, and potential therapeutic applications, helping the readers to critically evaluate the available literature and allowing them to consider new future possibilities. 

Carballo-Pedrares and Rey-Rico et al. [10] performed a revision of the current advances on the development and use of hydrogels as controlled gene delivery systems in regenerative medicine strategies, with particular emphasis on musculoskeletal tissue repair, cardiovascular tissue repair, wound healing, and neural tissue repair. Moreover, they presented and discussed various hydrogel systems that have been used to mediate the controlled delivery of different types of genetic material, such as pDNA, mRNA, siRNA and miRNA, underlining hydrogels as a growing and promising area of research needed for the development of efficient and safe future gene therapy-based treatments. 

On the other hand, Aulicino, Capin and Berger [11] focused their review manuscript on the current progress in the development of synthetic virus-derived nanosystems (SVNs), obtained by MultiBac baculovirus engineering, and in the implementation of CRISPR/Cas technologies into these vectors, conferring to SVNs the ability to introduce DNA into genomes with high precision. The authors described SVNs as nanosystems that are safe, readily customized, and that can be manufactured at large scale, constituting an attractive alternative to viral vectors, situated at the boundary between viral and non-viral delivery systems, for future genome engineering and gene therapy applications. 

Egorova and Kiselev et al. [12] developed iRGD ligand-conjugated cysteine-rich peptide nanocarriers (RGD1-R6) for targeted gene delivery to αvβ3 integrin-expressing primary UL cells. The authors performed the physicochemical characterization of nanosystems, and evaluated their cytotoxicity, transfection activity, specificity and capability to mediate the therapeutic strategy in target cells. The obtained data revealed that the DNA/RGD1-R6 nanocarriers exhibited specificity to αvβ3 integrin and have capacity to deliver a plasmid DNA encoding the herpes simplex virus thymidine kinase to UL cells, which when followed by GCV treatment resulted in a significant cell death effect, showing that the generated RGD1-R6 carrier can be a promising tool for gene therapy of uterine leiomyoma.

## References

[1] Guan G., Song B., Zhang J., Chen K., Hu H., Wang M., Chen D. (2019). An effective cationic human serum albumin-based gene delivery carrier containing nuclear localization signal. Pharmaceutics.

[2] Martínez-Negro M., Sánchez-Arribas N., Guerrero-Martínez A., Moyá M.L., Ilarduya C., Mendicuti F., Aicart E., Junquera E. (2019). A non-viral plasmid DNA delivery system consisting on a lysine-derived cationic lipid mixed with a fusogenic lipid. Pharmaceutics.

[3] Saher O., Lehto T., Gissberg O., Gupta D., Gustafsson O., Andaloussi S., Darbre T., Lundin K., Smith E., Zain R. (2019). Sugar and polymer excipients enhance uptake and splice-switching activity of peptide-dendrimer/lipid/oligonucleotide formulations. Pharmaceutics.

[4] Ibaraki H., Kanazawa T., Owada M., Iwaya K., Takashima Y., Seta Y. (2020). Anti-Metastatic Effects on Melanoma via intravenous administration of anti-NF-κB siRNA complexed with functional peptide-modified nano-micelles. Pharmaceutics.

[5] Nagachinta S., Bouzo B.L., Vázquez-Ríos A.J., Lopez R., de la Fuente M. (2020). Sphingomyelin-Based Nanosystems (SNs) for the development of anticancer miRNA therapeutics. Pharmaceutics.

[6] Taylor R.E., Zahid M. (2020). Cell penetrating peptides, novel vectors for gene therapy. Pharmaceutics.

[7] Roma-Rodrigues C., Rivas-García L., Baptista P.V., Fernandes A.R. (2020). Gene therapy in cancer treatment: Why going nano?. Pharmaceutics.

[8] Sato M., Inada M., Saitoh I., Watanabe S., Nakamura S. (2020). *piggy*Bac-Based non-viral in vivo gene delivery useful for production of genetically modified animals and organs. Pharmaceutics.

[9] Carvalho A.M., Cordeiro R.A., Faneca H. (2020). Silica-based gene delivery systems: From design to therapeutic applications. Pharmaceutics.

[10] Carballo-Pedrares N., Fuentes-Boquete I., Díaz-Prado S., Rey-Rico A. (2020). Hydrogel-based localized nonviral gene delivery in regenerative medicine approaches—An overview. Pharmaceutics.

[11] Aulicino F., Capin J., Berger I. (2020). Synthetic virus-derived nanosystems (SVNs) for delivery and precision docking of large multifunctional DNA circuitry in mammalian cells. Pharmaceutics.

[12] Egorova A., Shtykalova S., Selutin A., Shved N., Maretina M., Selkov S., Baranov V., Kiselev A. (2021). Development of iRGD-modified peptide carriers for suicide gene therapy of uterine leiomyoma. Pharmaceutics.

